# The Elusive *Troglobius brasiliensis* (Collembola: Paronellidae), a Critically Endangered Species Rediscovered and Redescribed in Its Habitat and Food Web

**DOI:** 10.1007/s13744-026-01427-1

**Published:** 2026-09-14

**Authors:** Douglas Zeppelini, Roniere Andrade de Brito, Aila Soares Ferreira, Bruna Carolline Honório Lopes, Nathan Paiva Brito, Misael Augusto de Oliveira Neto, Estevam Cipriano Araujo de Lima

**Affiliations:** 1 Lab de Sistemática de Collembola E Conservação, Instituto de Biologia Do Solo, Univ Estadual da Paraíba, João Pessoa, PB Brazil; 2 Programa de Pós-Graduação em Ciências Biológicas – Zoologia, Univ Federal da Paraíba, João Pessoa, PB Brazil; 3 Lab de Estudos Subterrâneos, Depto de Ecologia E Biologia Evolutiva, Univ Federal de São Carlos, São Carlos, SP Brazil; 4 Programa de Pós-Graduação em Ecologia e Recursos Naturais, Univ Federal de São Carlos, São Carlos, SP Brazil

**Keywords:** Cave fauna, Micro-endemic, Predation, Threatened species, Troglobite

## Abstract

*Troglobius brasiliensis* is a troglomorphic species known only from a single cave in the Brazilian rain forest. This is the first record of the species since its original collection in 1988 and subsequent formal description approximately 30 years ago. We present an integrative redescription of *T. brasiliensis* based on detailed morphological analysis with scanning electron microscopy. It is a coded redescription with a molecular marker (COI), comprehensive notes on its habitat. The species was found exclusively along the riverbank, restricted to a single sector of the aphotic zone, approximately 40 m from the main cave entrance; both adults and juveniles were observed, indicating a functional and reproductive population. The detailed morphological documentation presented here allows robust comparisons with all known species of *Troglobius*, and other members of family Paronellidae, contributing to refining species diagnosis and providing a standardized framework for future taxonomic and conservation studies involving subterranean fauna.

**Supplementary Information:**

The online version contains supplementary material available at https://doi.org/10.1007/s13744-026-01427-1.

## Introduction

*Troglobius brasiliensis* Palacios-Vargas & Zeppelini 1995 is a troglobitic species, restricted to a single cave in the Brazilian rain forest. The species was described from four slide-mounted specimens, deposited in the collection of the *Laboratorio de Ecología y Sistemática de Microartrópodos* at the *Universidad Nacional Autónoma de México* (LESM – UNAM). The type series is composed of three specimens, Holotype and two Paratypes, from the type locality, Caverna do Limoeiro, Medicilândia, Pará, Northern Brazil, and one Paratype from a different locality (Cave Gruta dos Paiva, Iporanga, São Paulo, Brasil), about 3000 km away, in the subtropical domains of Atlantic Forest. This is the first record of the species since it was collected in 1988, and the formal description 30 years ago (Palacios-Vargas and Zeppelini 1995).

The genus *Troglobius* Palacios-Vargas & Wilson 1990 is composed of four known species, mostly distributed in cave environments: *T. coprophagus* Palacios-Vargas & Wilson 1990 was described from limestone caves in Madagascar; *T. brasiliensis* occurs in two Brazilian caves: Limoeiro (Pará) and Gruta dos Paiva (São Paulo); *T. ferroicus* Zeppelini et al. 2014 was discovered in iron-ore cave in Minas Gerais, Brazil, and *T. albertinoi* Cipola et al. 2016, the only non-troglobitic species of the genus recorded so far, was found inside decaying logs in a forested area of the Brazilian Amazon (state of Amapá), possibly in association with ants or termites, as reported for other Cyphoderinae.

The original description of *T. brasiliensis* was based on a few specimens, with no information about its habitat and the distribution inside the cave or about the cave itself, other than locality. Furthermore, the specimen from *Gruta dos Paiva*, in Southeastern Brazil, is clearly a different species, as remarked in the original description and in a subsequent revision of this Paratype afterwards (Palacios-Vargas and Zeppelini 1995; Zeppelini et al. 2014).

The scarcity of information about the species, the ambiguous distribution and restriction to cave environment, associated with local threats to the cave system and the gap in the conservation efforts, rendered to *T. brasiliensis* the status of Critically Endangered (CR) in the Brazilian fauna List of Threatened Species (Brasil 2014: MMA Decree No. 443/2014). In order to understand the threats and determine an appropriate conservation program for the species, two expeditions were made to find the animal in its type locality (2022), and to search for records in caves and surface in the nearby region (2023) to determine its actual distribution.

As results, we will present a detailed morphological analysis under scanning electron microscopy (SEM), a coded redescription with a molecular marker (COI), and a comprehensive description of its habitat, role in the food web, and distribution. By doing so, we seek to refine its taxonomic diagnosis, clarify its distribution, and provide essential information to support future studies and conservation strategies for the species.

## Material and methods

Detailed methodological procedures can be accessed in supplementary information (S1).

### Study area

**Fig. 1:**
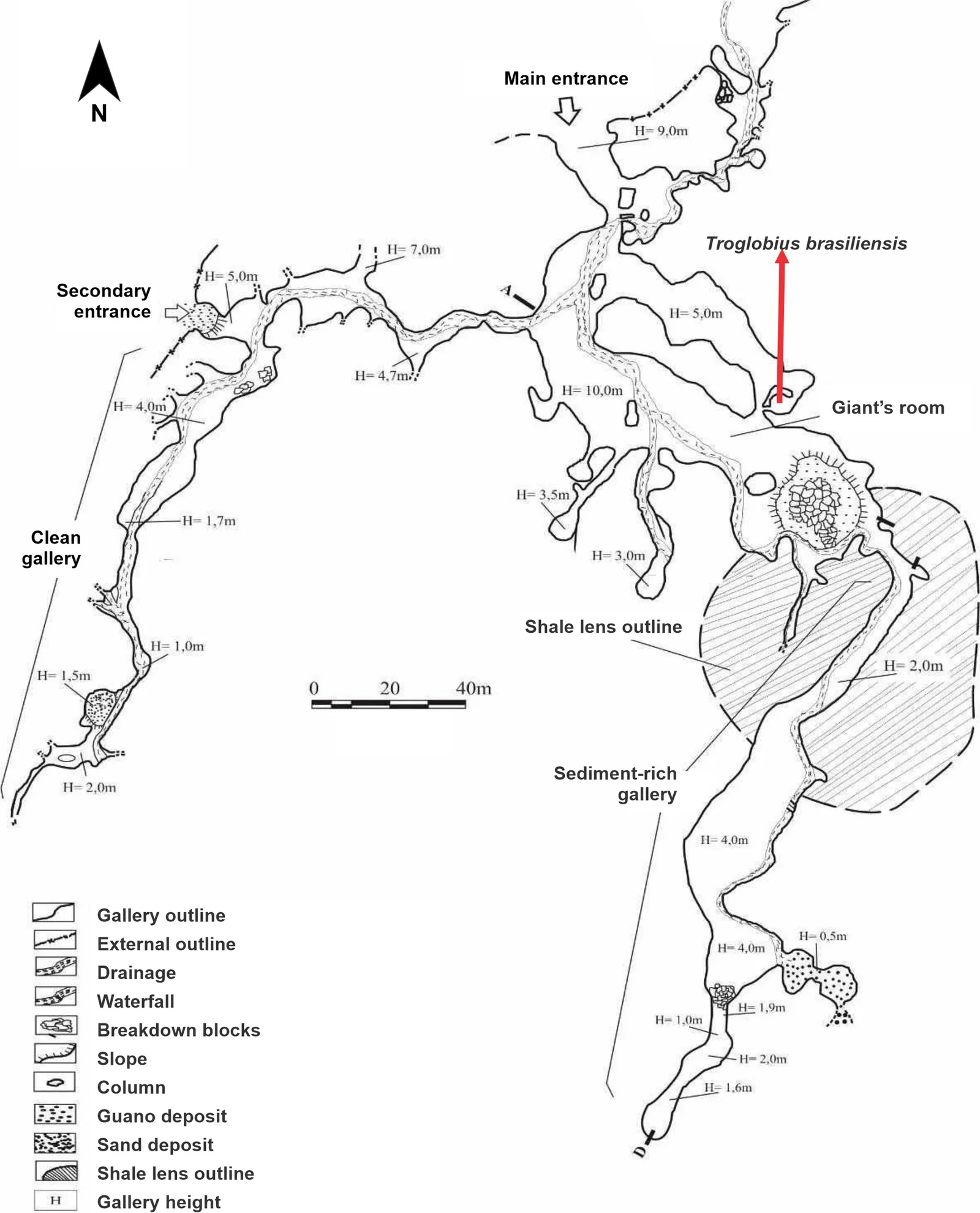
Floor plan of the Limoeiro Cave, Medicilândia/PA, type locality of the species *Troglobius brasiliensis*, highlighting the occurrence and capture site (adapted from GEP 2001)

**Fig. 2:**
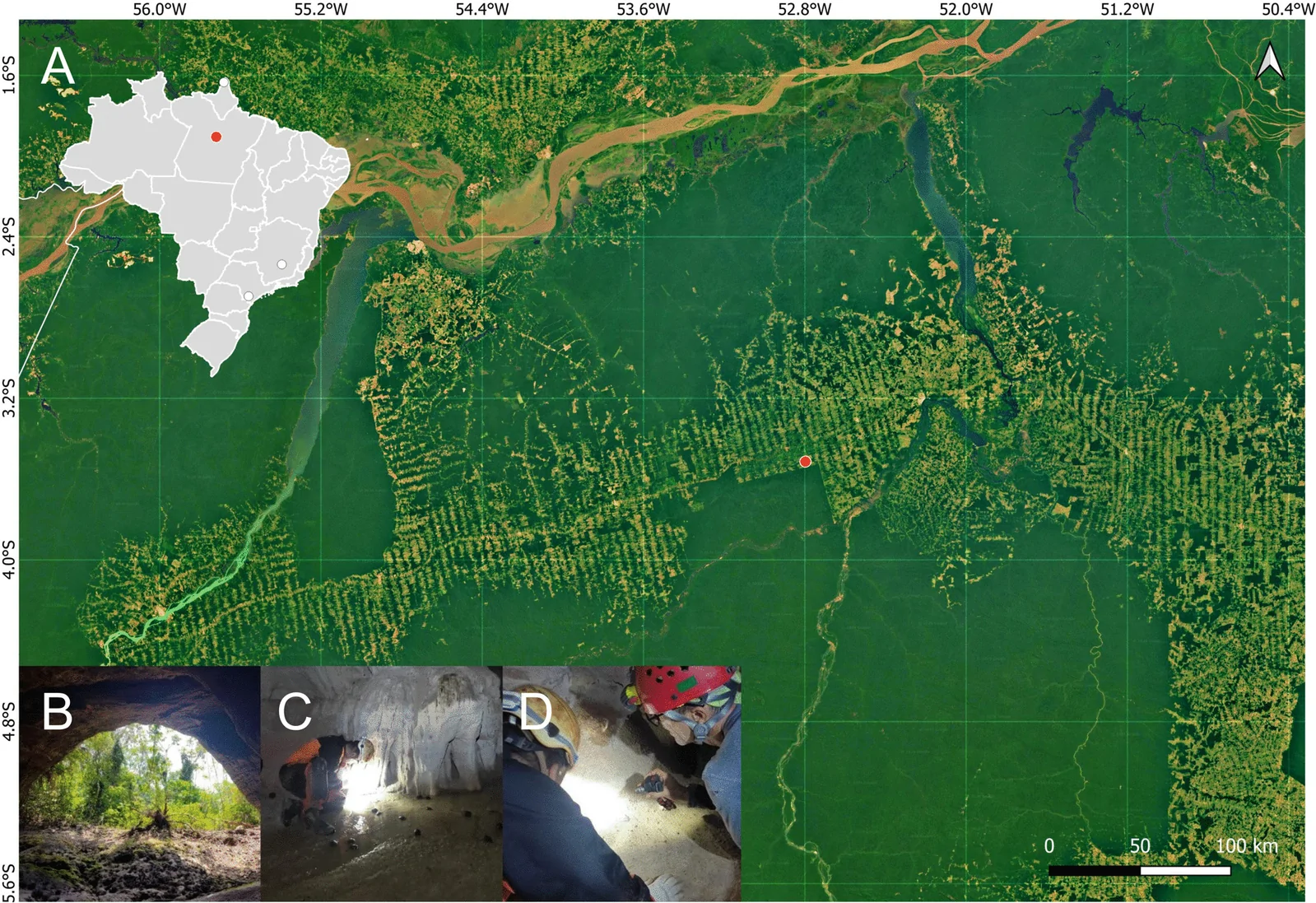
Type locality and habitat of *Troglobius brasiliensis.* **A** Geographic location of Limoeiro Cave (3°30′47.87″S, 52°47′51.83″W), Medicilândia, Pará State, Brazil. The red dot indicates the cave location; inset shows the position of Pará State within Brazil. Note the erosion of the forest, showing the “fishbone” pattern; **B**–**D** cave environment and sampling area: **B** cave entrance; **C** riverbank inside the cave where the species was found; **D** precise spot where most specimens were collected

The two expeditions (2022 and 2023) to the Limoeiro cave (3°30′47.87″S/52°47′51.83″W) in Medicilândia, a municipality in the Northern state of Pará (Figs. 1 and 2), were carried out by the Institute for Forestry Development and Biodiversity of the State of Pará (IDEFLOR–Bio), with the support of the Institute of Soil Biology – State University of Paraiba (IBS – UEPB).

The cave is situated on a private property, mostly dedicated to livestock; however, the surroundings of the cave are relatively well conserved, with a dense rain forest cover. The cave is somewhat remote, and the absence of roads and the property’s vigilance maintains the cave partially isolated. Nevertheless, vestiges of hunting activities were seen in the cave entrance.

The lithology is composed by the sedimentary Amazonic Basin, in friable sandstone of the Maecuru formation. The cave is about 1200 m long in the two main galleries, oriented WNW-ESE. The cave has three entrances, two wide open (the main entrance is 15 m wide and 9 m high; the second is 5 × 5 m), and the third one is the river resurgence that carved a wide room with a rather low and narrow opening to the outside (Freire et al. 2019).

Other six caves were sampled for *T. brasiliensis*, as follows: *Gruta Cama de Vara* cave (Altamira/PA: 3°18′42.5″S/52°14′47.9″W), *Abrigo do Igarapé* cave (Vitória do Xingu/PA: 3°13′46.4″S/52°00′27.6″W), *Pedra da Cachoeira* cave (Altamira/PA: 3°19′14.5″S/52°19′52.9″W), *Sugiro/Roncador* cave (Altamira/PA: 3°17′55.5″S/52°14′06.0″W), *Abrigos Assurini* cave (Altamira/PA: 3°15′05.0″S/52°11′16.4″W), and *Planaltina* cave (*Brasil Novo*/PA: 3°22′40.1″S/52°34′31.4″W).

A revision of the collection of Collembola of the *Museu Paraense Emílio Goeldi* (MPEG) included 561 records from different localities, collected since 1960. Additionally, samples of Collembola from the Laboratory of Zoology of the *Universidade federal do Pará* (UFPA – Altamira) were revised.

### Coded description

For the taxonomic description, we used the coded taxonomic description method proposed by Zeppelini et al. 2024 modified by Oliveira and Zeppelini 2024. The method consists of presenting the general morphology in a coded matrix, based on a list of characters with all observed conditions for each character in the list. The morphological features are presented in a pictorial matrix, side by side at the same scale. The images in the matrix are obtained mainly by SEM, or the best available resolution. The description is presented as a matrix column, containing codes (1,2,3) for each and every character described, sided by the corresponding morphologic illustration. The illustration can be either photographic images or schematic line drawings, depending on the feature. The presentation results in a descriptive template.

As an example of the readability of the character condition definition in the character list, we can take one character in the list below (Table 1):

**Table 1:** Character list used in the integrative coded taxonomic descriptions, including morphological structures, characters, and observed character states (97 characters, corresponding to the DELTA matrix)

Character				Observed condition(s)
DNA COI				**1** – Genbank: PV843549 **2** – different entry
Pigments				**1** – absent. **2** – present
Body color				**1** – white. **2** – pale yellow
Head	Segment I	Eye	Eye number (EN)	**1** – 0 + 0. **2** – different combination
		PAO	Post-antennal organ (PAO)	**1** – absent. **2** – present
		Clipeal	Clypeal area I (CI)	**1** – 1 + 1 2(1). **2** – different combination
			Clypeal area II (CII)	**1** – 1 + 1 2(1). **2** – different combination
			Clypeal area III (CIII)	**1** – 2 + 2 4(1). **2** – different combination
			Clypeal area IV (CIV)	**1** – 1 + 1 + 1 3(1). **2** – different combination
		Labral	Labral formula (a + m + p + pl)	**1** – 4 + 5 + 5 + 4 18(1). **2** – different combination
	Segment II	Antenal	Antennal area chaetotaxy (ANTC)	**1** – 8 + 8 10(13)6(29). **2** – different combination
	Segmento IV	Mandibular	Mandibular area chaetotaxy (MD)	**1** – 10 + 1 + 10 21(2). **2** – different combination
	Segment V	Maxillary area	Maxillary area chaetotaxy dorsal (MX D)	**1** – 13 + 13 24(2)2(12a). **2** – different combination
			Maxillary area chaetotaxy dorsolateral and ventral (MX L + V)	**1** – 25 + 25 48(1)2(12b). **2** – different combination
		Maxillary lobe	Maxillary distal area (LMX D)	**1** – 4 + 4 6(1)2(3). **2** – different combination
			Maxillary basal area (LMX B)	**1** – 1 + 1 2(1). **2** – different combination
	Segment VI	Labial	Labial area chaetotaxy dorsal and ventral (LB D + V)	**1** – 2 + 2 4(1). **2** – different combination
			Labial chaetae alongside the ventral groove (LBVG)	**1** – 5 + 5 10(1). **2** – different combination
			Labial triangle total chaetae (LBT)	**1** – 10 + 10 20(1). **2** – different combination
			Labial proximal chaetae and papillae total chaetae (LBP)	**1** – 24 + 24 24 + 24 10(1)2(2)10(5)2(6)18(7)6(8). **2** – different combination
Body	Thorax	Thorax I	Thorax I (ThI)	**1** – 0 + 0. **2** – different combination
		Thorax II	Thorax II (ThII)	**1** – 14 + 14 26(2)2(9). **2** – different combination
		Thorax III	Thorax III (ThIII)	**1** – 7 + 7 12(2)2(10). **2** – different combination
			Thorax III ventral (VM)	**1** – 0 + 0. **2** – different combination
	Abdomen	Abdomen I	Abdomen I (AbdI)	**1** – 6 + 6 10(2)2(9). **2** – different combination
		Abdomen II	Abdomen II (AbdII)	**1** – 13 + 13 6(2)2(10)4(12a)4(16)10(17). **2** – different combination
		Abdomen III	Abdomen III (AbdIII)	**1** – 18 + 18 6(2)2(9)2(10)6(12a)4(16)12(17)4(18). **2** – different combination
		Abdomen IV	Abdomen IV (AbdIV)	**1** – 55 + 55 38(2)22(10)6(12a)28(16)12(17)4(18). **2** – different combination
			Abdomen IV posterior area total pseudopores (AbdIV PSP)	**1 –** 3 + 3 6(29)** 2** – different combination
			Abdomen IV area parafurcal total chaetae (AbdIV PF)	**1** – 24 + 24 48(16). **2** – different combination
		Abdomen V	Abdomen V (AbdV)	**1** – 15 + 15 8(2)6(10)4(16)12(18). **2** – different combination
		Abdomen VI	Abdomen VI (AbdVI)	**1** – 34 + 2 + 34 40(2)30(16)**. 2** – different combination
Head apendagges	Antenna	Antenna I	Antenna I whorl A total chaetae (ANTI A)	**1** – 10 1(2)9(13). **2** – different combination
			Antenna I whorls -I 0 and + I total chaetae (ANTI −0 +)	**1 –** 35 3(1)8(2)20(13)4(20)**. 2 –** different combination
			Antenna I whorl B total chaetae (ANTI B)	**1** – 2 2(19). **2** – different combination
		Antenna II	Antenna II whorl A total chaetae (ANTII A)	**1** – 11 1(2)7(13)2(18)1(20). **2** – different combination
			Antenna II whorls -I 0 and + I total chaetae (ANTII −0 +)	**1** – 92 11(2)58(13)19(18)2(20)2(21). **2** – different combination
			Antenna II whorl B total chaetae (ANTII B)	**1** – 3 1(2)2(19). **2** – different combination
		Antenna III	Antenna III whorl A total chaetae (ANTIII A)	**1** – 10 4(13)2(20)3(21). **2** – different combination
			Antenna III whorls -I 0 and + I total chaetae (ANTIII −0 +)	**1** – 140 19(2)85(13)34(18)2(21). **2** – different combination
			Antenna III whorl B total chaetae (ANTIII B)	**1** – 8 8(13). **2** – different combination
		Antenna IV	Antenna IV A total chaetae (ANTIV A)	**1** –324 157(13)61(18)4(20)12(21)57(22)1(24)32(25) **2** – different combination
			Antenna IV B total chaetae (ANTIV B)	**1** – 9 9(13). **2** – different combination
Body apendagges	Legs	Subcoxa	Subcoxa I total chaetae (SCXI)	**1** – 7 7(14). **2** – different combination
			Subcoxa II total chaetae (SCXII)	**1** – 11 4(14)7(16). **2** – different combination
			Subcoxa III total chaetae (SCXIII)	**1** – 10 6(14)3(16)1(29). **2** – different combination
		Coxa	Coxa I total chaetae (CXI)	**1 –** 12 1(11)10(15)1(29). **2 –** different combination
			Coxa II total chaetae (CXII)	**1 –** 13 1(11)8(14)4(15). **2 –** different combination
			Coxa III total chaetae (CXIII)	**1 –** 12 1(11)4(14)6(15)1(29). **2 –** different combination
		Trochanter	Trochanter I total chaetae (TRI)	**1** – 18 2(4)15(1)1(16). **2** – different combination
			Trochanter II total chaetae (TRII)	**1** – 14 2(4)9(14)2(16)1(29). **2** – different combination
			Trochanter III total chaetae (TRIII)	**1** – 27 8(14)2(16)16(26). **2** – different combination
		Femur	Femur I total chaetae (FEI)	**1** – 55 1(4)44(13)10(16). **2** – different combination
			Femur II total chaetae (FEII)	**1** – 62 1(4)53(13)8(16). **2** – different combination
			Femur III total chaetae (FEIII)	**1** – 41 17(13)8(16)16(26). **2** – different combination
		Tibiotarsi	Tibiotarsus I total chaetae (TibI)	**1** – 95 79(2)15(16)1(31). **2** – different combination
			Tibiotarsus II total chaetae (TibII)	**1** – 110 100(2)9(16)1(31). **2** – different combination
			Tibiotarsus III total chaetae (TibIII)	**1** – 83 70(2)12(16)1(31). **2** – different combination
		Pretarsusi	Pretarsus I pretarsal chaetae (PTI)	**1** – 2 2(2). **2** – different combination
			Pretarsus II pretarsal chaetae (PTII)	**1** – 2 2(2). **2** – different combination
			Pretarsus III pretarsal chaetae (PTIII)	**1** – 2 2(2). **2** – different combination
		Unguis	Unguis I inner tooth basal: posterior and anterior (UI b.p + b.a)	**1 –** pair equal. **2 –** pair unequal. **3 –** absent
			Unguis I inner tooth: medial (UI m.t)	**1 –** normal. **2 –** absent. **3 –** reduced
			Unguis I inner tooth: apical (UI a.t)	**1 –** normal**. 2 –** absent**. 3 –** reduced
			Unguis II inner tooth: basal posterior and anterior (UII b.p + b.a)	**1 –** pair equal. **2 –** pair unequal. **3 –** absent
			Unguis II inner tooth: medial (UII m.t)	**1 –** normal. **2 –** absent. **3 –** reduced
			Unguis II inner tooth: apical (UII a.t)	**1 –** normal**. 2 –** absent**. 3 –** reduced
			Unguis III inner tooth: basal posterior and anterior (UIII b.p + b.a)	**1 –** pair equal. **2 –** pair unequal. **3 –** absent
			Unguis III inner tooth: medial (UIII m.t)	**1 –** normal. **2 –** absent. **3 –** reduced
			Unguis III inner tooth: apical (UIII a.t)	**1 –** normal**. 2 –** absent**. 3 –** reduced
		Unguiculus	Unguiculus I antero-internal lamella (UCI a.i)	**1 –** smooth. **2 –** serrate
			Unguiculus I antero-external lamella (UCI a.e)	**1 –** smooth. **2 –** serrate
			Unguiculus I postero-internal lamella (UCI p.i)	**1 –** smooth. **2 –** serrate
			Unguiculus I postero-external lamella (UCI p.e)	**1 –** with outer tooth. **2 –** smooth
			Unguiculus II antero-internal lamella (UCII a.i)	**1 –** smooth. **2 –** serrate
			Unguiculus II antero-external lamella (UCII a.e)	**1 –** smooth. **2 –** serrate
			Unguiculus II postero-internal lamella (UCII p.i)	**1 –** smooth. **2 –** serrate
			Unguiculus II postero-external lamella (UCII p.e)	**1 –** with outer tooth. **2 –** smooth
			Unguiculus III antero-internal lamella (UCIII a.i)	**1 –** smooth. **2 –** serrate
			Unguiculus III antero-external lamella (UCIII a.e)	**1 –** smooth. **2 –** serrate
			Unguiculus III postero-internal lamella (UCIII p.i)	**1 –** smooth. **2 –** serrate
			Unguiculus III postero-external lamella (UCIII p.e)	**1 –** with outer tooth. **2 –** smooth
	Collophore	Anterior side	Anterior side total chaetae (AS)	**1** – 2 + 2 4(14). **2** – different combination
		Lateral flap	Lateral flap total chaetae (LF)	**1** – 3 + 3 6(1). **2** – different combination
		Posterior side	Posterior side total chaetae (PS)	**1 –** 8 + 2 + 8 10(2)4(4)4(11)**. 2 –** different combination
	Tenaculum	Corpus Tenacular	Tenaculum rami (TR)	**1** – 4 + 4 teeth. **2** – different combination
			Tenaculum corpus (a + p) (TC a + p)	**1** – 1 1(13). **2** – different combination
	Furcula	Manubrium	Anterior manubrium total chaetae (MNA)	**1** – 1 + 1 2(13). **2** – different combination
			Posterior manubrium total chaetae (MNP)	**1** – 67 + 67 130(13)4(29). **2** – different combination
		Dens	Dental total chaetae: basal (DNB)	**1** – 6 6(13). **2** – different combination
			Dental total chaetae: Central (DNC)	**1 –** 69 10(13)56(27)3(29)**. 2 –** different combination
			Dental total chaetae: apical (DNA)	**1** – 3 3(13). **2** – different combination
			Dens posterior total chaetae: internal row (DPI)	**1** – 23 23(27). **2** – different combination
			Dens posterior total chaetae: medial row (DPM)	**1** – 25 4(13)18(27)3(29). **2** – different combination
			Dens posterior total chaetae: external row (DPE)	**1** – 21 3(13)18(27). **2** – different combination
		Mucro	Mucro inner lamella (MUI)	**1** – serrate with 14 teeth. **2** – smooth
			Mucro outer lamella (MUO)	**1** – serrate with 14 teeth. **2** – smooth

- (Structure) Labral chaetotaxy
- (Character) Labral formula a + m + p + pl:
- (Observed condition) 1– 4+5+5+4 9(1)9(4)
- (Observed condition) 2 – different combination

In the description template, there will be a code (1 or 2). The meaning of the description 4 + 5 + 5 + 4 9(1)9(4) is that there is a total of 18 chaetae, distributed in the rows: a – 4 chaetae, m – 5 chaetae, p – 5, pl – 4, where nine chaetae are the type (1) in the pictorial matrix, and nine chaetae are type (4) in the matrix. This procedure avoids the use adjectives to describe some morphologic features (as different types of chaetae), reducing the ambiguity.

Descriptive diagnosis was automatically generated using the FreeDELTA Editor through Descriptions → Natural Language (CONFOR directives). The set of characters retained corresponds to those traditionally employed in species-level diagnoses within the genus. The DELTA files are available at https://doi.org/10.5281/zenodo.21890449.

### Assessment of morphology and homology

Specimens were preserved in 70% ethanol and prepared following standard protocols for Collembola for light microscopy (Jordana and Arbea 1997). For observations under SEM, specimens were dried with a critical point dryer and coated in gold. Figures and plates were produced and assembled using CorelDRAW Graphics Suite 2020 for Windows.

Specimens are deposited in the *Coleção de Referência de Fauna de Solo, Instituto de Biologia do Solo*, at the *Universidade Estadual da Paraíba* (CRFS–IBS–UEPB) under the following inventory numbers: specimens in slides #24703, #24914, #24915, #24916, #24917, #24918/CRFS/UEPB, and specimen in stub, #M01/CRFS/UEPB. Available at the GBIF (https://www.gbif.org/occurrence/search?q=troglobius%20brasiliensis&dataset_key=8cf92f87-eaee-4d1c-96a9-49c3f5998351).

Homology was inferred in accordance with the ontogenetic and morphologic studies for the group, as follows: antennal sensillae and chaetotaxy followed Deharveng (1983) and Nayrolles (1991); dorsal chaetotaxy of head after Nunes and Bellini (2018) and Cipola et al. (2016); labial chaetotaxy followed Gisin (1967) and Zhang and Pan (2020); labial papillae, maxillary palp, and basolateral and basomedial labial fields as in Fjellberg (1998); clypeal chaetotaxy followed Yoshii and Suhardjono (1992); labral chaetotaxy followed Cipola et al. (2014); body chaetotaxy after Szeptycki (1979), with additions of Zhang et al. (2019) and Oliveira et al. (2023); specialized chaetae (S-chaetae) as in Zhang and Deharveng (2015).

### Molecular analysis

A single specimen of *T. brasiliensis* was used for DNA extraction, with a non-destructive method that preserves the cuticle. The voucher was mounted in a semi-permanent slide for optical microscopy, to certify the identification, and was deposited at the CRFS/UEPB (#24703/CRFS/UEPB). The 710-bp fragment of the mitochondrial Cytochrome C Oxidase Subunit I (COI) gene was amplified using the forward primer LCO-1490 (5′-ggtcaacaaatcataaagatattgg-3′) and the reverse primer HC0-2198 (5′-taaacttcagggtgaccaaaaaatca-3′) (Folmer et al. 1994). The PCR product purification and Sanger sequencing were performed by ACTGene Molecular Analysis (https://actgene.com.br). The obtained forward and reverse sequences were analyzed and edited using Geneious Prime software (version 2025.1.2). The obtained sequences were analyzed by Basic Local Alignment Search Tool (BLAST) and deposited in the GenBank (#PV843549). The COI sequence homology was analyzed, along with sequences from 52 specimens of the family Paronellidae, available and published at the National Center for Biotechnology Information (NCBI), including *T. albertinoi*, the only species of the same genus available. Sequences were aligned with codon MACSE (version 2.07) algorithm (Ranwez et al. 2018). Best-fitting nucleotide substitution model was selected under Bayesian Information Criterion (BIC) using ModelFinder implemented in IQ-TREE (version 2.0.7, Nguyen et al. 2015; Kalyaanamoorthy et al. 2017). Phylogenetic relationships were inferred using maximum likelihood (ML) in IQ-TREE under the selected model, with branch support assessed using 1000 ultrafast bootstrap replicates (UFBoot) and 1000 SH-like approximate likelihood ratio test replicates (SH-aLRT). All analyses were automated using scripts implemented in Python version 3.12.3. (https://www.python.org).

## Results

### COI identification and positioning

**Fig. 3:**
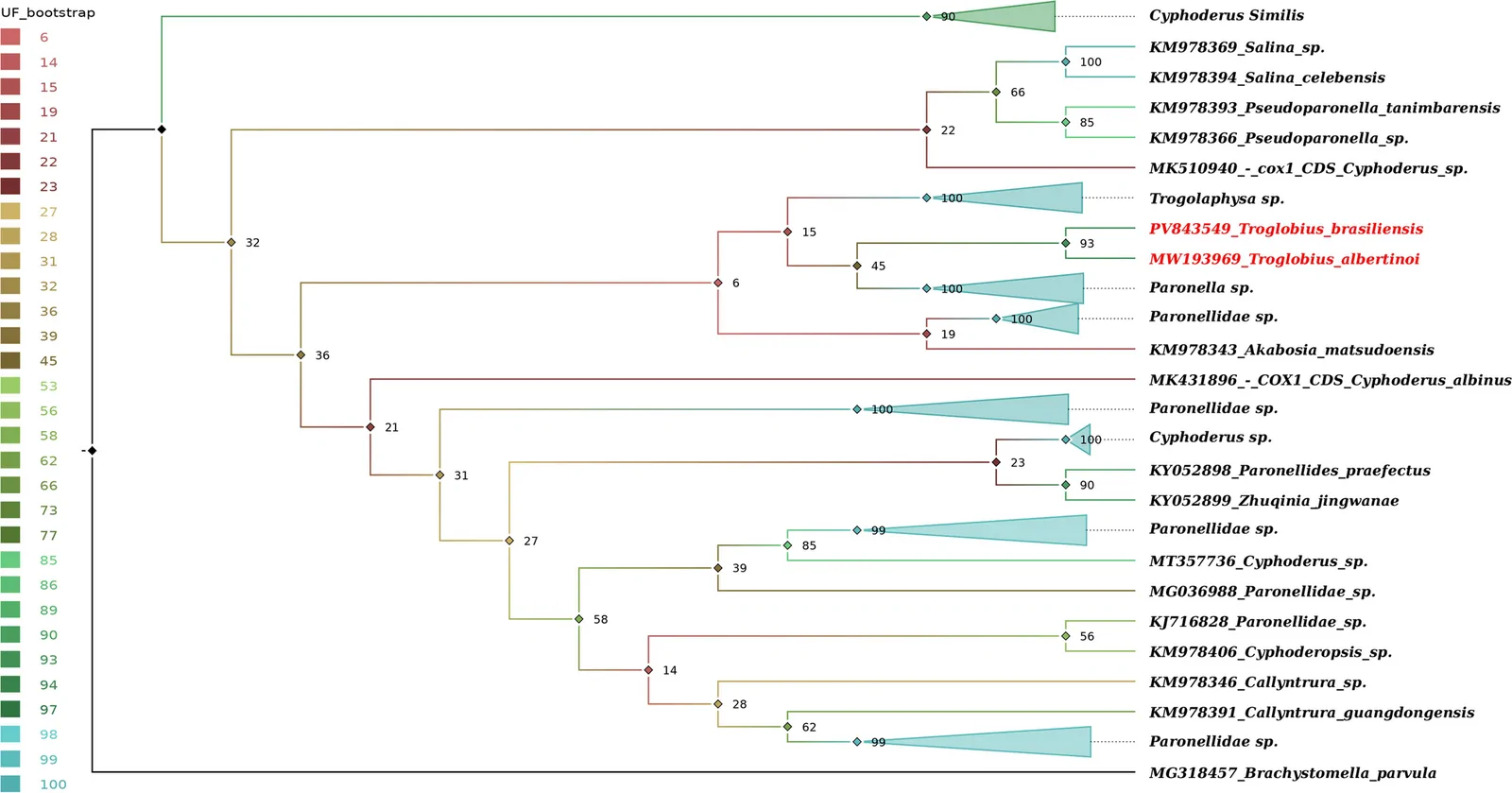
Maximum likelihood phylogenetic tree includes 54 COI sequences from Paronellidae taxa obtained from GenBank and sequence the *Troglobius brasiliensis* generated in this study. Node labels indicate ultrafast bootstrap (UFBoot) support values and color-coded according to the scale shown (values ranging from 6 to 100). Collapsed branches represent groups of highly similar sequences. The clade containing the *Troglobius* species is highlighted, showing its close phylogenetic relationship with the species of the same genus within the family Paronellidae

The consensus tree with 53 terminal clades clustered the two species of the genus, *T. brasiliensis* and *T. albertinoi*, together as sister species, indicating the monophyly of the genus (Fig. 3). The same tree shows that the genus *Troglobius* is close to *Trogolaphysa* and *Paronella* as part of the family Paronellidae, tribe Paronellini. The consensus tree and alignment log files are available in the repository, together with the scripts, at publications: https://doi.org/10.5281/zenodo.21890449.

### Integrative coded taxonomy

Entomobryomorpha Börner, 1913

Familia Paronellidae Börner, 1913

Subfamily Cyphoderinae Börner, 1913 sensu Soto-Adames et al. 2008

Genus *Troglobius* Palacios-Vargas & Wilson 1990

urn:lsid:zZoobank.org:act:059322ED-EB50-40ED-8E8E-B7FAC9E20FDB

GenBank PV843549

**Fig. 4:**
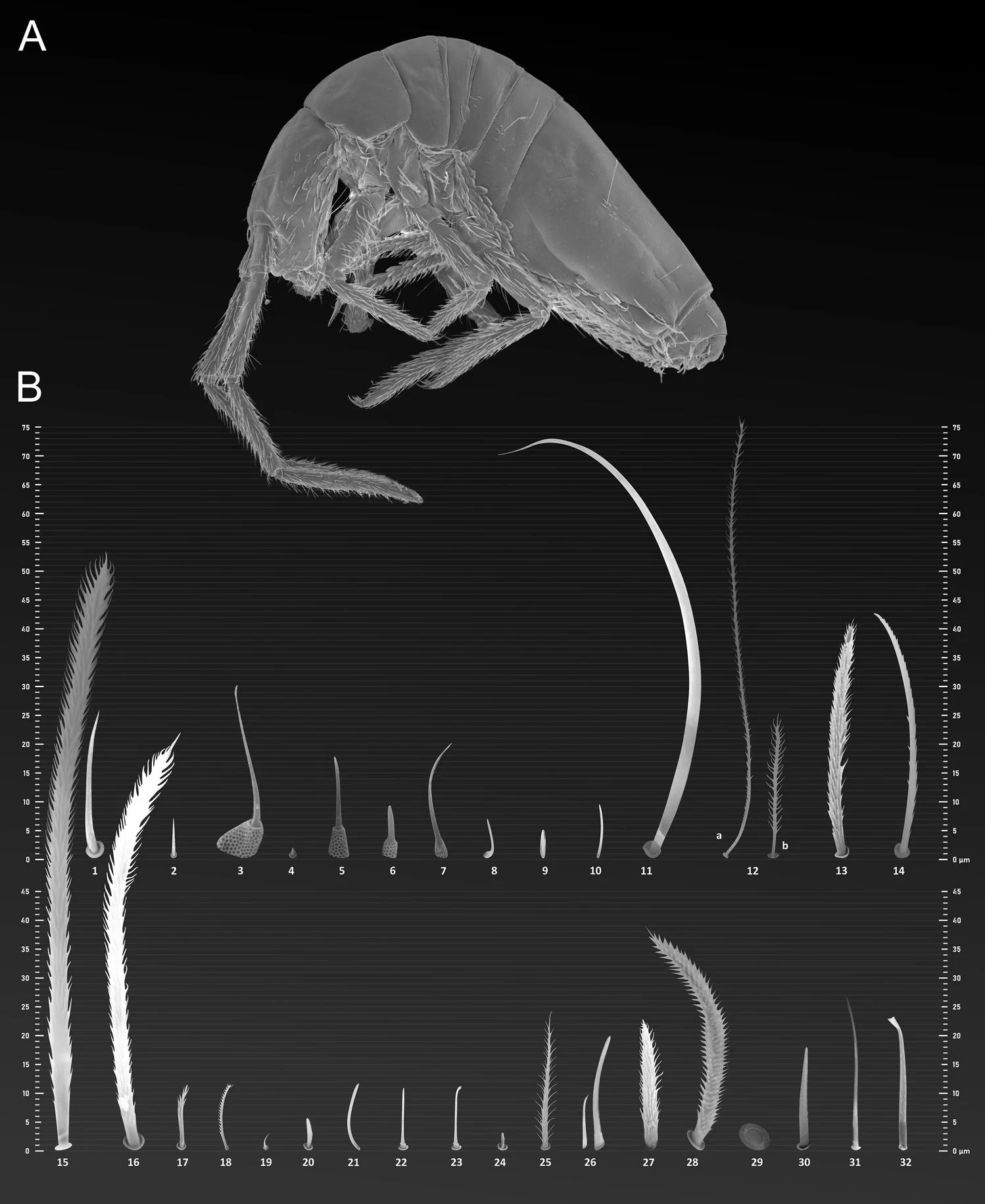
Pictorial matrix of Paronellidae. **A** Habitus; **B** morphological Bank—a collection of different chaetae found along the body in *Troglobius brasiliensis* and *Cyphoderus* sp. The chaetae are represented in their shapes and sizes; therefore, a given shape and size represented in the bank may occur in different parts of the body: 1—normal chaeta (usually all body); 2—microchaeta (usually all body); 3—macrosensillum with papilla; 4—drop-like microchaeta; 5—mesosensillum with papillae (e.g., mesosensillum of the labial palp–A); 6—rod-shaped mesosensillum (e.g., labial palp, finger shape lateral process –l.p.); 7—curved mesosensillum inserted in a triangular socket (e.g., mesosensillum labial palp—ai); 8—chaeta (e.g., mesosensillum labial H); 9—S-microchaetae (ms); 10—S-chaetae (sens); 11—smooth macrochaeta (occurring on coxae); 12—bothriotricha; 13—heavily ciliated chaeta; 14—ciliated mesochaeta; 15—heavily ciliated macrochaeta; 16—heavily ciliated macrochaeta with a spinelike apex; 17—branched microchaeta (often around bothriotricha); 18—short-ciliated chaeta; 19—microsensillum; 20—thick and blunt macrosensillum (present on the antenna); 21—thick and acuminate mesosensillum; 22—elongate rod-shaped mesosensillum (often on Ant.IV); 23—elongate rod-shaped mesosensillum flattened at the tip (often on Ant.IV); 24—thick and blunt microsensillum (often subapically on Ant. IV); 25—long-ciliated chaeta; 26—spinelike chaeta (often on metatrochanteral organ); 27—thick heavily ciliated chaeta (often on the dens the *Troglobius*); 28—thick heavily ciliated mesochaeta (often on the dens the *Cyphoderus*)***;***29—pseudopore; 30—ciliate spinelike chaeta (often on the tibiotarsus of *Cyphoderus*); 31—bristle-like acuminate (tenent hair); 32—capitate bristle-like (tenent hair)

**Fig. 5:**
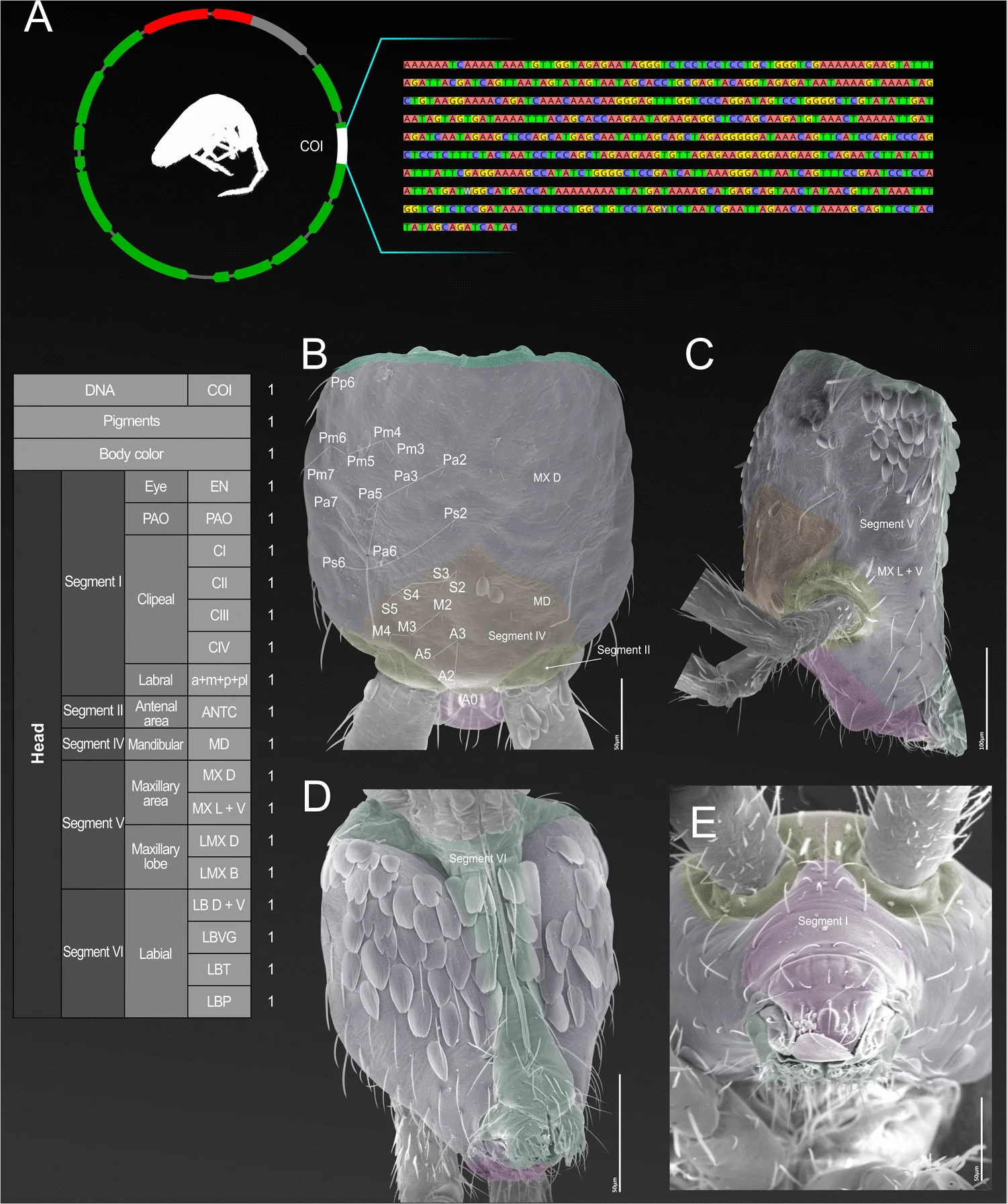
Troglobius brasiliensis. **A** COI sequence. **B**–**E** SEM images. Cephalic chaetotaxy and descriptive table. **B** Dorsal view of the cephalic capsule; **C** dorsolateral view of the cephalic capsule; **D** ventral view of the cephalic capsule; **E** anterior (frontal) view of the cephalic capsule

**Fig. 6:**
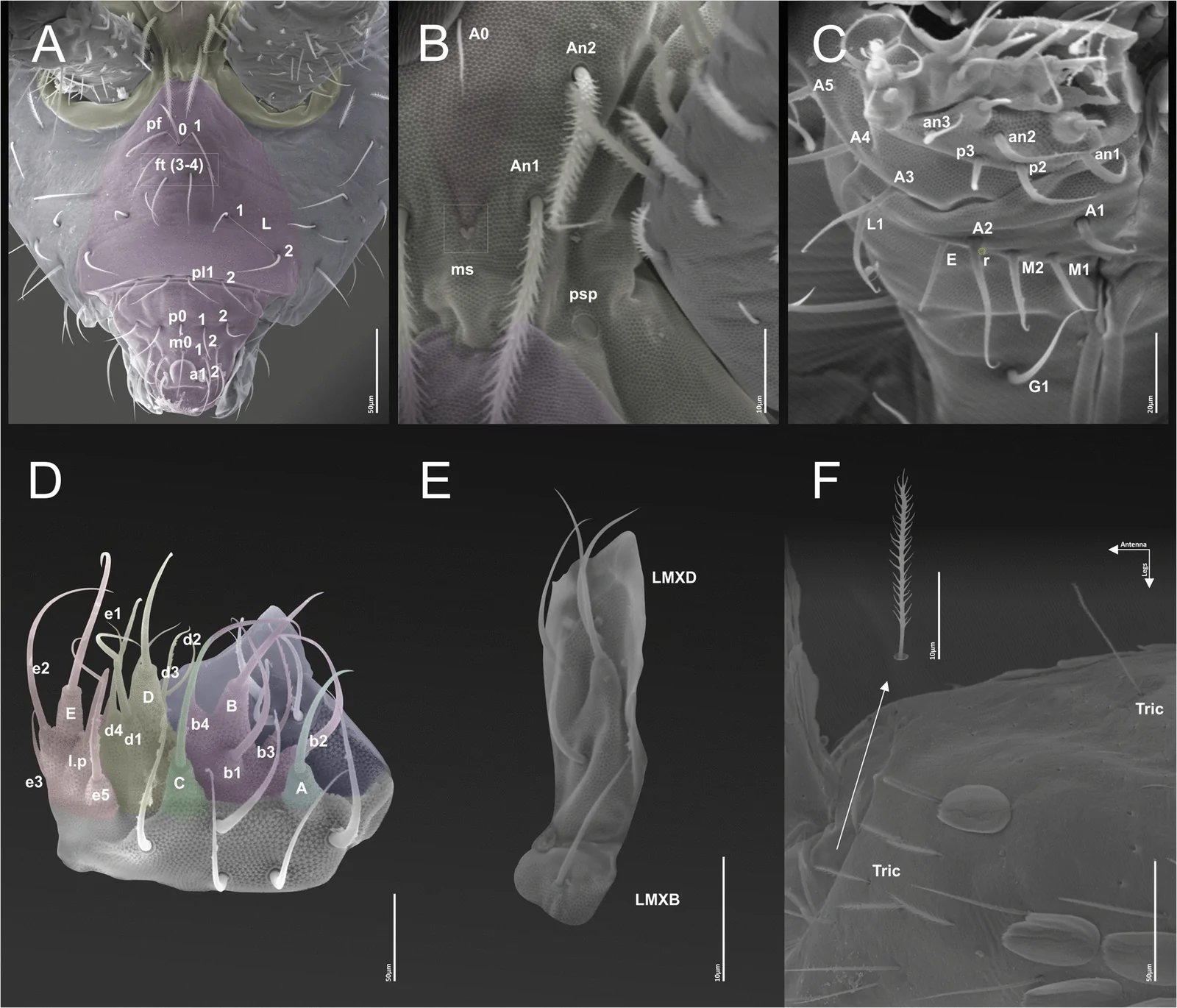
Troglobius brasiliensis. SEM images. Cephalic chaetotaxy. **A** Segment I: clypeal and labral areas; **B** segment II: detailed view of the sensillum and pseudopores; **C** segment VI: labial triangle, labial papillae and proximal chaetae, top view; **D** segment VI: labial papillae, lateral view; **E** segment V: maxillary lobe; **F** details of cephalic trichobothria

**Fig. 7:**
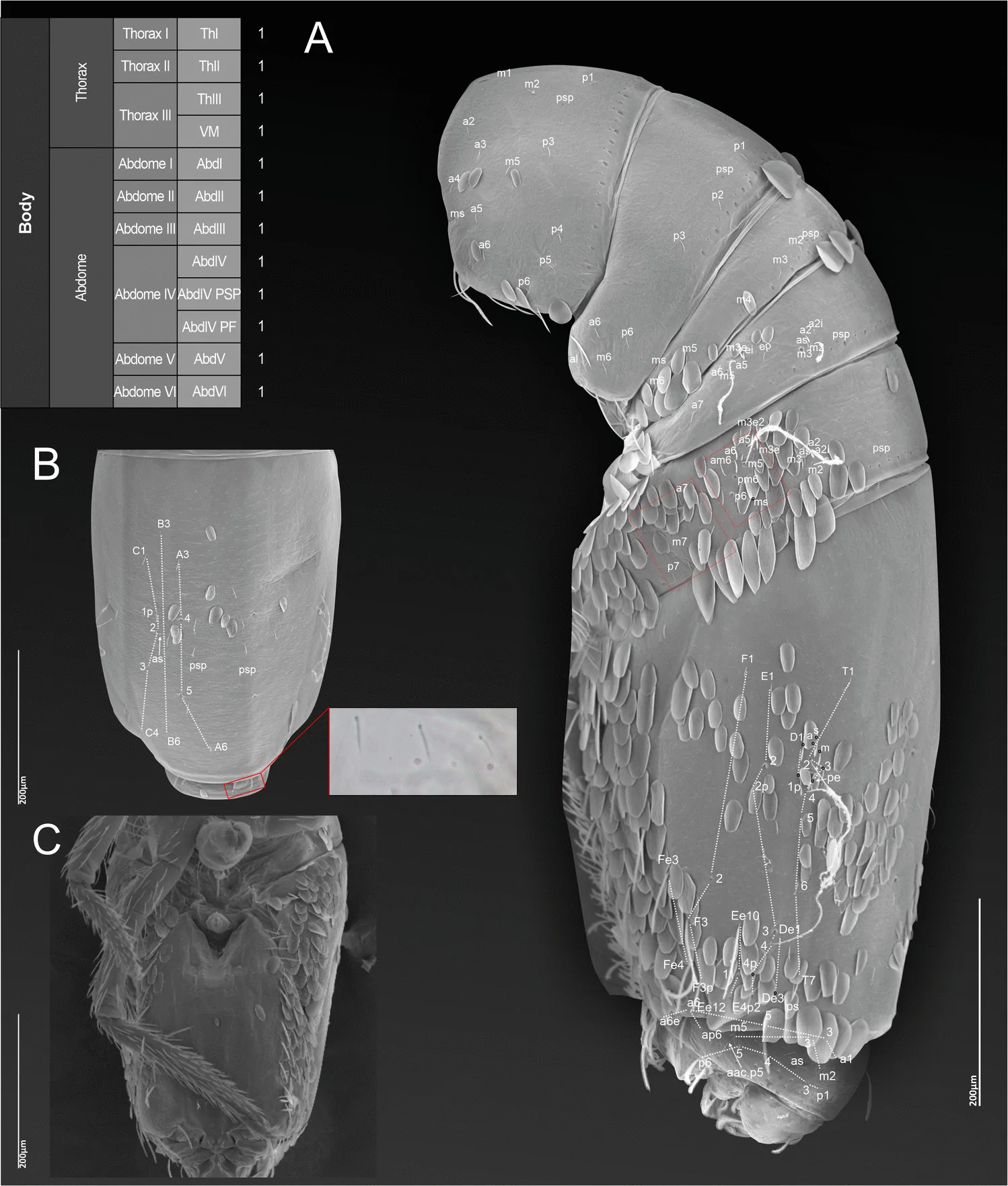
Troglobius brasiliensis. SEM images. Body chaetotaxy and descriptive table. **A** Body in lateral view; **B** abdomen IV with details of pseudopores; **C** abdomen parafurcal area (AbdIV PF)

**Fig. 8:**
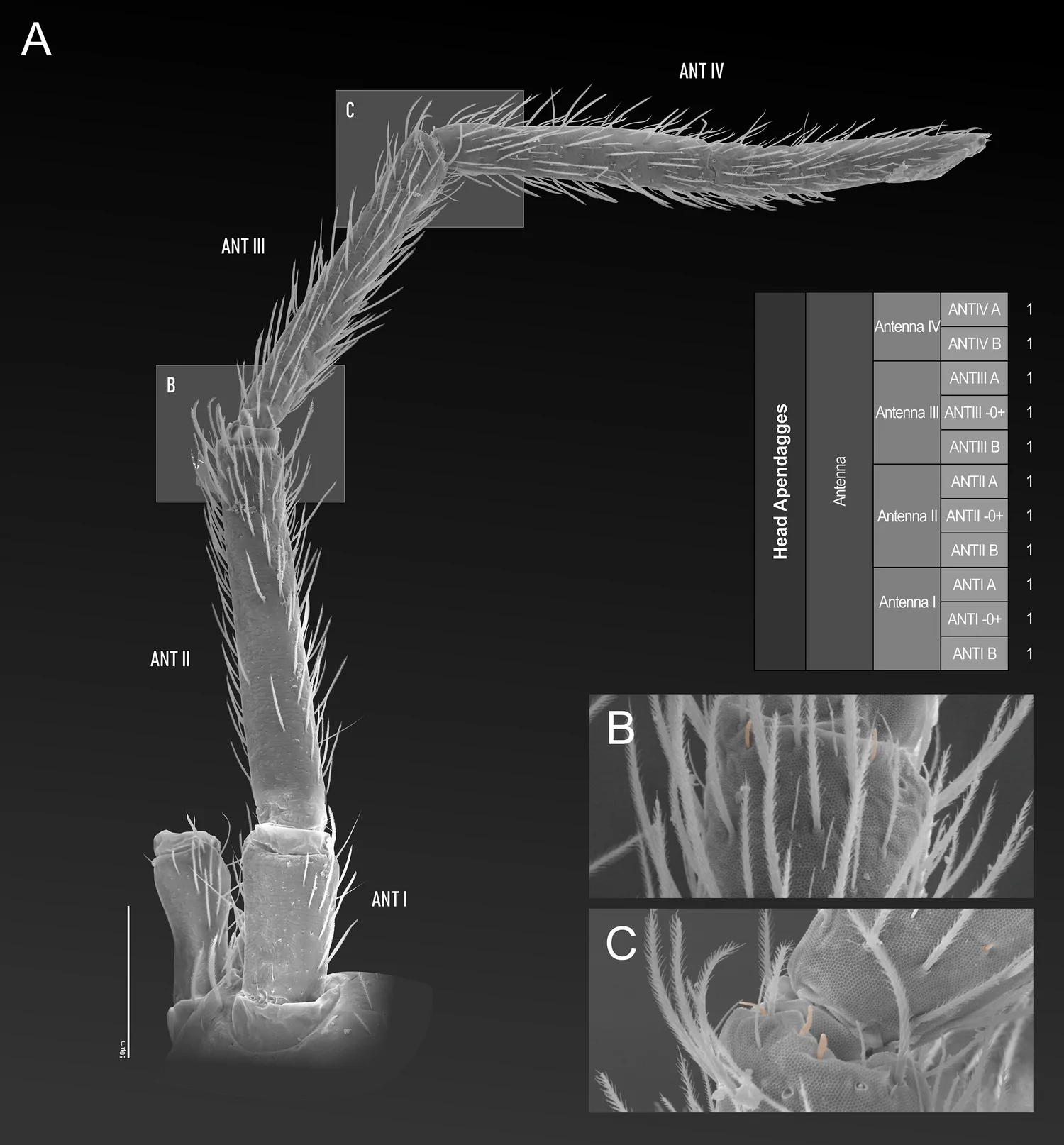
Troglobius brasiliensis. SEM images. Antennal chaetotaxy and descriptive table. **A** Entire antennal; **B** Ant. II apex view; **C** Detail of the sensilla of the Ant III apical organ

**Fig. 9:**
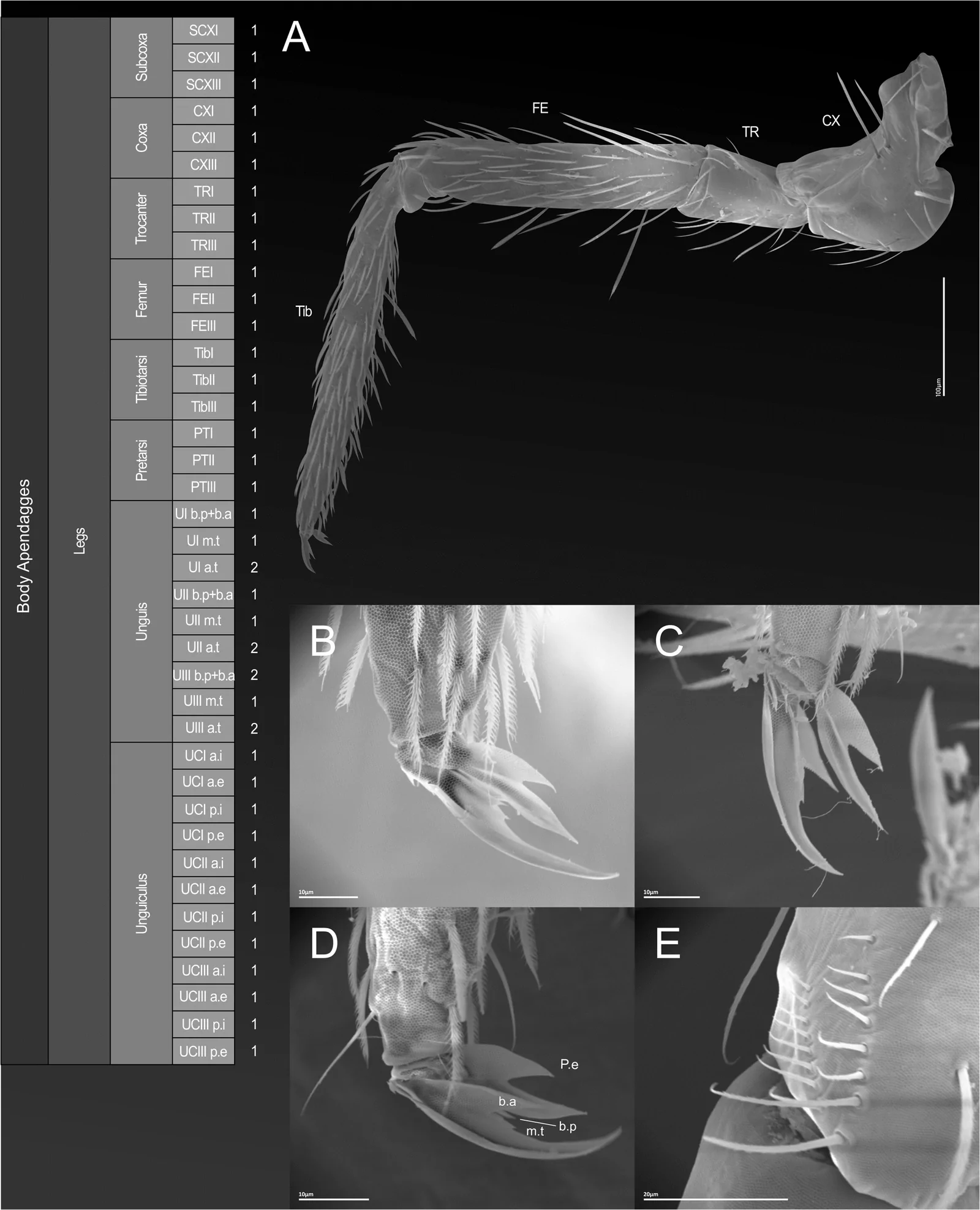
Troglobius brasiliensis. SEM images. Leg chaetotaxy and descriptive table. **A** Leg I; **B** empodial complex I; **C** empodial complex II; **D** empodial complex III; **E** metatrochanteral organ

**Fig. 10:**
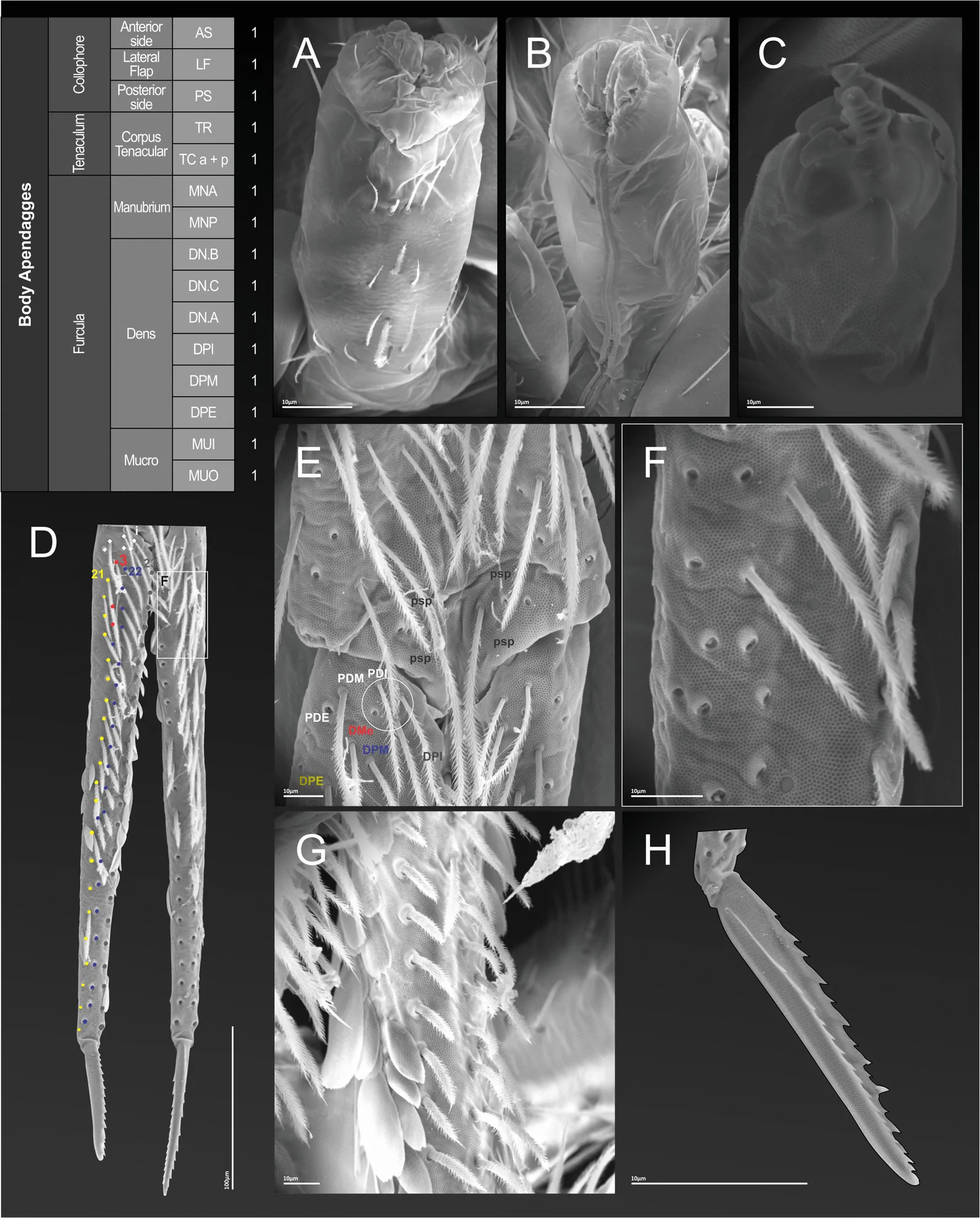
Troglobius brasiliensis. SEM images. Abdominal appendages chaetotaxy. **A** Collophore: lateral flap (LF) and posterior side (PS); **B** collophore: anterior side (AS); **C** tenaculum; **D** furcula; **E** manubrial plate and base of the dens; **F** details of dens with pseudopores; **G** details of the dental chaetotaxy pattern, chaeta 27 (pictorial matrix of Paronellidae); **H** mucro

***Troglobius brasiliensis*** Palacios-Vargas & Zeppelini 1995 (Table 1, Figs. 4, 5, 6, 7, 8, 9, and 10)

**Examined material**. Six specimens: In slides (#24914, #24917/CRFS/UEPB): Brazil, Pará State, Medicilândia Municipality, Limoeiro Cave, 03°30′43.1″S 52°47′49.1″W, 10 Dec. 2022, Brito, R. A. Zeppelini, D. coll. Additional slides (#24915, #24918, #24916/CRFS/UEPB) and stubs for SEM (#M01/CRFS/UEPB): same data, except 18 Aug. 2023.

**Distinctive characters**. Abdomen II chaeta **p5** absent; Abdomen III with macrochaetae **p6** and **pm6**; Abdomen IV with macrochaetae **A4** and **A5**, and 3 + 3 pseudopores. Trochanteral organ with 16 chaetae. Unguis with paired basal teeth (**b.a** and **b.p**) and one unpaired median tooth (**m.t**). Collophore with lateral flaps 3 + 3 chaetae and posterior side with 8+2+8 chaetae. Dens posterior side with three rows of modified thick chaetae, with 23 chaetae in the internal row, 22 in the median row, and 21 in the external row. Mucro with both internal and external lamellae bearing 14 teeth.

**Descriptive diagnosis**. DNA COI Genbank: PV843549 (Fig. 5). Pigments (PI) absent. Body color (BC) White. **Head** (Figs. 5 and 6). Eye number (EN) 0 + 0. Clypeal area (CI) 1 + 1 2(1). Clypeal area (CII) 1 + 1 2(1). Clypeal area III (CIII) 2 + 2 4(1). Clypeal area (CIV) 1 + 1 + 1 3(1). Labral formula (a + m + p + pl) 4 + 5 + 5 + 4 18(1). Maxillary area chaetotaxy dorsal (MX D) 13 + 13 24(2)2(12a). Labial chaetae, alongside the ventral groove (LBVG) 5 + 5 10(1). Labial triangle total chaetae (LBT) 10 + 10 20(1). Labial proximal chaetae and papillae total chaetae (LBP) 24 + 24 10(1)2(2)10(5)2(6)18(7)6(8). Thorax I (ThI) 0 + 0. **Body** (Fig. 7). Thorax II (ThII) 14 + 14 26(2)2(9). Thorax III (ThIII) 7 + 7 12(2)2(10). Thorax III ventral (VM) 0 + 0. Abdomen I (AbdI) 6 + 6 10(2)2(9). Abdomen II (AbdII) 3 + 13 6(2)2(10)4(12a)4(16)10(17). Abdomen III (AbdIII) 18 + 18 6(2)2(9)2(10)6(12a)4(16)12(17)4(18). Abdomen IV (AbdIV) 55 + 55 38(2)22(10)6(12a)28(16)12(17)4(18). Abdomen IV posterior area total pseudopores (AbdIV PSP) 3 + 3 6(29). Abdomen IV area parafurcal total chaetae (AbdIV PF) 24 + 24 48(16). Abdomen V (AbdV) 15 + 15 8(2)6(10)4(16)12(18). Abdomen VI (AbdVI) 34 + 2 + 34 40(2)30(16). **Antenna** (Fig. 8). Antenna I whorl A total chaetae (ANTI A) 10 1(2)9(13). Antenna I whorls -I 0 and + I total chaetae (ANTI −0 +) 35 3(1)8(2)20(13)4(20). Antenna I whorl B total chaetae (ANTI B) 2 2(19). Antenna II whorl A total chaetae (ANTII A) 11 1(2)7(13)2(18)1(20). Antenna II whorls −I 0 and +I total chaetae (ANTII −0+) 92 11(2)58(13)19(18)2(20)2(21). Antenna II whorl B total chaetae (ANTII B) 3 1(2)2(19). Antenna III whorl A total chaetae (ANTIII A) 10 4(13)2(20)3(21). Antenna III whorls −I 0 and +I total chaetae (ANTIII −0+) 140 19(2)85(13)34(18)2(21). Antenna III whorl B total chaetae (ANTIII B) 8 8(13). Antenna IV A total chaetae (ANTIV A) 324 157(13)61(18)4(20)12(21)57(22)1(24)32(25). Antenna IV B total chaetae (ANTIV B) 9 9(13). **Legs** (Fig. 9). Subcoxa I total chaetae (SCXI) 7 7(14). Subcoxa II total chaetae (SCXII) 11 4(14)7(16). Subcoxa III total chaetae (SCXIII) 10 6(14)3(16)1(29). Coxa I total chaetae (CXI) 12 1(11)10(15)1(29). Coxa II total chaetae (CXII) 13 1(11)8(14)4(15). Coxa III total chaetae (CXIII) 12 1(11)4(14)6(15)1(29). Trochanter I total chaetae (TRI) 18 2(4)15(14)1(16). Trochanter II total chaetae (TRII) 14 2(4)9(14)2(16)1(29). Trochanter III total chaetae (TRIII) 27 8(14)2(16)16(26). Femur I total chaetae (FEI) 55 1(4)44(13)10(16). Femur II total chaetae (FEII) 62 1(4)53(13)8(16). Femur III total chaetae (FEIII) 41 17(13)8(16)16(26). Unguis III inner tooth: basal posterior and anterior (UIII b.p + b.a) pair unequal. Unguis III inner tooth: medial (UIII m.t) normal. Unguis III inner tooth: apical (UIII a.t) absent. Unguiculus II postero-external lamella (UCII p.e) with outer tooth. Unguiculus III antero-internal lamella (UCIII a.i) smooth. Unguiculus III antero-external lamella (UCIII a.e) smooth. Unguiculus III postero-internal lamella (UCIII p.i) smooth. Unguiculus III postero-external lamella (UCIII p.e) with outer tooth. **Collophore** (Fig. 10A and B). Anterior side total chaetae (AS) 2 + 2 4(14). Lateral flap total chaetae (LF) 3 + 3 6(1). Posterior side total chaetae (PS) 8 + 2 + 8 10(2)4(4)4(11). **Furcula** (Fig. 10D–H). Anterior manubrium total chaetae (MNA) 1 + 1 2(13). Posterior manubrium total chaetae (MNP) 67 + 67 130(13)4(29). Dental total chaetae: basal (DNB) 6 6(13). Dental total chaetae: Central (DNC) 69 10(13)56(27)3(29). Dental total chaetae: apical (DNA) 3 3(13). Dens posterior total chaetae: internal row (DPI) 23 23(27). Dens posterior total chaetae: medial row (DPM) 25 4(13)18(27)3(29). Dens posterior total chaetae: external row (DPE) 21 3(13)18(27). Mucro inner lamella (MUI) serrate with 14 teeth. Mucro outer lamella (MUO) serrate with 14 teeth.

**Distribution**. *Troglobius brasiliensis* is restricted to the Limoeiro cave, in Medicilândia, Pará, at the Northern region of Brazil (Fig. 2), in a vast area called *Volta Grande do Xingú*, East portion of the Amazon rain forest. The Koeppen climate classification is Af, Equatorial rain forest, fully humid (Kottek et al. 2006). Although museum collections spanning over five decades of records from surface and cave environments, and the recent active search within caves and leaf litter on the region, no record of *T. brasiliensis* has been found, further supporting its status as an endemic troglobite restricted to a single cave.

**Remarks**. The present redescription of *T. brasiliensis* is based exclusively on specimens collected from the type locality (Limoeiro Cave, Pará). *Troglobius brasiliensisis* unique within the genus in having a mucro with 14 teeth on both the internal and external lamellae, whereas *T. albertinoi* and *T. ferroicus* have one tooth on the inner lamella, and it is absent in *T. coprophagus*. The species further differs from *T. albertinoi* by the presence of Abd. III with chaeta **p6** as a macrochaeta (microchaeta in *T. albertinoi*) and Abd. IV with 3 + 3 posterior pseudopores (1 + 1 in *T. albertinoi*). It differs from *T. coprophagus* by unguis unpaired inner tooth (absent in *T. coprophagus*) and dens with 23 chaetae in the internal row (9–10 in *T. coprophagus*). It also differs from *T. ferroicus* by Abd. I with chaeta **m2** present (absent *T. ferroicus*) and trochanteral organ with 16 chaetae (34 in *T. ferroicus*).

**Habitat**. The species is restricted to a single sandstone cave (Fig. 1), from which emerges a stream that flows out from the cave, not providing resources to the cave habitat. The cave is spacious; there are many bat colonies and large bat guano deposits piled on the floor, mainly frugivorous and insectivorous. There are associated fauna to the bat guano piles, mainly crickets (*Endecous* sp.) and cockroaches (*Blaberus* sp.), even though they are not usually seen sharing guano piles, instead they show discrete distribution. The walls and floor of the cave are habitats for an abundance of scorpions, amblypygids, pseudoscorpions, spiders, and harvestmen. However, we did not find any *T. brasiliensis* in those habitats, despite the detailed search effort. The species was found exclusively at the riverbank, in a relatively small area of the aphotic zone, about 40 m away from the main entrance of the cave, in a somewhat isolated lateral chamber, resulting in a micro-endemic distribution within a single cave.

Two additional species of Collembola were found in the cave with disjunct distributions; *Troglobius brasiliensis* is the only troglobite found so far, whereas the other two are typically epigeic species and must be found in the leaflitter or epiedaphic habitats.

**Population and food webs**. The species shows low abundance, usually about 20 to 30 individuals, was observed each visit to the site. The presence of adult females, adult males, and juveniles is indication of a viable and breeding population. Contrary to expectations, the species was not observed on bat guano or any sort of debris; instead, they were found exclusively on clean sandstone along the riverbank. The entire habitat is moist and mostly covered by a thin film of water. Since no visible food source was observed, it is presumed that *T. brasiliensis*, primarily feed on microalgae, fungi and bacterial biofilm (Fig. 2C and D).

On the other hand, its role as prey was recorded (see video https://doi.org/10.5281/zenodo.21890449). A nymph of *Spelaeochernes altamirae* (Arachnida, Pseudoscorpionida) was observed preying upon an adult specimen, while holding it with the pedipalp. No adults of pseudoscorpion or any other predator were observed interacting with *T. bralisiensis*, which may indicate that the species plays an important role as food source for immature stages, of at least some, of the predators that feed and reproduce in the cave (particularly the troglophiles and troglobites). This seems to be consequence of its tiny size and low agonistic response other than escape, jumping by means of the furcula. Also, the dead individuals may serve as eventual food item for juveniles of small predators.

The whole habitat structure is strictly connected to the stream that runs across the species occupation grounds; a change in the water regime can disturb the complete riverbank in a single event, and drive *T. brasiliensis* and, likely, the related predatory species to extinction in a drastically short period of time.

## Conclusions

The most relevant findings are the unequivocal definition of the distribution of *T. brasiliensis*, which is a micro-endemic troglobite confined to a single chamber inside the Limoeiro cave, with a discrete and defined habitat. Also, despite some vestige of hunting activities in the entrance of the cave, we verified the suitable and conserved conditions of the area where the cave is located; however, we cannot disregard the pressure around the whole area, occupied with activities that can impact the cave system and the water regime of the region, resulting in a direct and potentially drastic effect on the species and the whole fauna inside the cave.

The detailed morphologic study and integrative coded description of this elusive species will allow comparisons with all the species of the genus *Troglobius* and the family Paronellidae, setting a new standard of detail for future taxonomic descriptions, enhanced even more by the molecular marker.

Finally, the finding of a reproductive population and predatory interaction are evidence of a healthy and functional ecosystem, with elements connected to each other in a highly delicate interdependent food web.

## Supplementary Information

Below is the link to the electronic supplementary material.

ESM 1 (DOCX 8.01 KB)

## Declarations

### Data Transparency

Sequence data used in this study are all available via GenBank. The morphological matrix and associated scripts are available in the repository: https://doi.org/10.5281/zenodo.21890449.
