# Supplementary material for: The Elusive Troglobius brasiliensis (Collembola: Paronellidae), a Critically Endangered Species Rediscovered and Redescribed in Its Habitat and Food Web
Source: Neotrop Entomol. 2026 Sep 14;55(1):86. doi: 10.1007/s13744-026-01427-1 (PMC13574854; doi:10.1007/s13744-026-01427-1)
Supplement: Supplementary file 1 [file 13744_2026_1427_MOESM1_ESM.docx]

**The elusive *Troglobius brasiliensis* (Collembola: Paronellidae), a critically endangered species rediscovered and redescribed in its habitat and food web**

Douglas Zeppelini^1,2^, Roniere Andrade de Brito^1^, Aila Soares Ferreira^1*^, Bruna Carolline Honório Lopes^1^, Nathan Paiva Brito^1,2^, Misael Augusto de Oliveira Neto^3,4^, Estevam Cipriano Araújo de Lima^1^

^1^Laboratório de Sistemática de Collembola e Conservação, Instituto de Biologia do Solo, Universidade Estadual da Paraíba, João Pessoa, PB, Brasil

^2^Programa de Pós-Graduação em Ciências Biológicas – Zoologia, Universidade Federal da Paraíba, João Pessoa, PB, Brasil

^3^Laboratório de Estudos Subterrâneos, Departamento de Ecologia e Biologia Evolutiva, Universidade Federal de São

Carlos, São Carlos, SP, Brasil

⁴Programa de Pós-Graduação em Ecologia e Recursos Naturais, Universidade Federal de São Carlos, São Carlos, SP, Brasil

*Correspondent Author: [aillasoares@gmail.com](mailto:aillasoares@gmail.com)

**Supplementary Information 1 (S1).** Detailed methodological procedures for scanning electron microscopy (SEM) and DNA extraction and amplification protocols for Collembola

**Scanning Electron Microscope**

For the study under Scanning Electron Microscope (SEM), the specimens were dehydrated by graded series of ethanol, dried in a critical point dryer model Autosamdri-931 by Tousimis, metallized with system for electron microscopes, HP Desk V model by Denton Vacuum and observed in SEM by Tescan Vega III model.  Previously the samples were hydrated for cleaning and rinsing, by a graded ethanol treatment with immersion for 20 minutes in 90% ethanol, then transferred to 60% ethanol, to 30% ethanol, and distilled water. After this initial process, the specimens were gradually dehydrated using 30%, 60%, and 90% ethanol, with 20 minutes in each concentration. Finally, the samples were placed in 99.8% ethanol and transferred to the critical point dryer.

**Pre-treatment and non-destructive DNA extraction**

A single specimen of *Troglobius brasiliensis* was placed in a 1.5 mL microtube and subjected to pre-treatment with 20 µL of Proteinase K and 400 µL of DNA/RNA Shield™ (Quick-DNA/RNA™ Viral MagBead Kit, Zymo Research) for 24 hours at 56 °C prior to DNA extraction. After brief centrifugation, the supernatant was transferred to a sterile microcentrifuge tube for DNA extraction, and 100 µL of 99.5% ethanol was added to the original tube to preserve the specimen for subsequent morphological analysis. The DNA voucher was mounted on a semi-permanent slide for observation under optical microscopy, deposited at the CRFS/UEPB (inventory number #24703/CRFS/UEPB).

DNA extraction from the supernatant recovered after pre-treatment was performed using the Quick-DNA/RNA™ Viral MagBead Kit (Zymo Research), following the manufacturer's instructions. An aliquot of 5 μL from each extraction was analyzed by 1% agarose gel electrophoresis to verify DNA integrity, and DNA was quantified using a NanoDrop One spectrophotometer (Thermofisher).

**PCR amplification and sequences analysis**

The 710-pb fragment of the mitocondrial Cytochrome C Oxidase Subunit I (COI) gene was amplified using the forward primer LCO-1490 (5'-ggtcaacaaatcataaagatattgg-3') and the reverse primer HC0-2198 (5'-taaacttcagggtgaccaaaaaatca-3') (Folmer *et al.* 1994). Polymerase chain reaction (PCR) mixtures (25 µL) consisted of 0.2 mM of each dNTP, 0.4 µM of each primer, 1 U of GoTaq® G2 Hot Start Polymerase (Promega), and 40 ng of *T. brasiliensis* genomic DNA. PCR was conducted with a Thermal Cycler (Applied Biosystems) with an initial cycle of 3 min at 94°C, followed by 30 cycles of denaturation at 94°C for 45 sec, annealing at 47°C for 45 sec, and extension at 72°C for 1 min. Cycles were finished by an elongation step of 7 min at 72°C. The PCR products were verified by 1 % agarose gel electrophoresis stained with Safer Dye (k9-16C) using a 100 bp DNA ladder (Ludwig) as a size reference.

PCR product purification and Sanger sequencing were performed by ACTGene Molecular Analysis ([https://actgene.com.br](https://actgene.com.br/)). The obtained forward and reverse sequences were analyzed and edited using Geneious Prime software (version 2025.1.2). They were then processed, and low-quality regions with more than a 5% chance of error per base at the ends were annotated. Thus, a consensus sequence was defined through an assembly using the forward and reverse sequences as a template. The obtained sequences were analyzed by Basic Local Alignment Search Tool (BLAST) and deposited in the GenBank.
